# Seasonal Variation and Genetic Evaluation of Needle Catechin Content in Half-Sib Families of *Pinus taeda*

**DOI:** 10.3390/plants15111666

**Published:** 2026-05-29

**Authors:** Jimeng Sun, Ling Wang, Tianyi Liu, Jiexian Luo, Chengcheng Gao, Shaowei Huang, Xueli Zhang, Jiawen Yu, Fenfen Liu, Liangyu Cao, Yan Zhang, Chenggong Liu

**Affiliations:** 1 College of Forestry and Landscape Architecture, South China Agricultural University, Guangzhou 510642, China; sjm376131292@163.com (J.S.); wl1985141861@126.com (L.W.); jxluo@stu.scau.edu.cn (J.L.); shwhuang@scau.edu.cn (S.H.); yjw42124@outlook.com (J.Y.); caoliangyu2026@163.com (L.C.); 2 Guangdong Key Laboratory for Innovative Development and Utilization of Forest Plant Germplasm, Guangzhou 510642, China; 3 State Key Laboratory of Tree Genetics and Breeding, Research Institute of Forestry, Chinese Academy of Forestry, Beijing 100091, China; gaocc@caf.ac.cn (C.G.); zxl961109@163.com (X.Z.); fen@caf.ac.cn (F.L.); zhangyan85913@163.com (Y.Z.); 4 Liaoning Provincial Research Institute of Poplar, Gaizhou 115213, China

**Keywords:** *Pinus taeda*, catechin content, BLUP model, selection strategy, family effect

## Abstract

The biosynthesis and accumulation of plant secondary metabolites are tightly regulated by environmental fluctuations, serving as a crucial interface mediating plant–environment interactions. Nevertheless, the phenotypic instability of secondary metabolism-related traits induced by environmental variability has hampered the precise breeding of stress-resistant cultivars. *Pinus taeda* is an key timber tree species in southern China, and its foliar catechins exhibit substantial stress-resistant potential. However, phenotypic variation driven by seasonal changes has limited the germplasm innovation and genetic selection of this species. In this study, 54 half-sib families of *P. taeda* were used as experimental materials. Combined with near-infrared spectroscopy (NIRS) and the BLUP model, we systematically analyzed the seasonal variation characteristics, genetic parameters of catechin content (CC), and genetic gains under different breeding strategies across four seasons. Our results demonstrated that family and season had extremely significant effects on CC (*p* < 0.001), whereas the season × family interaction effect was not significant, indicating that the genetic expression of CC is stable across seasons. CC was higher in spring and winter but lower in summer and autumn; specifically, the mean CC in summer was 47% lower than the peak value in spring (26.95 ± 0.46 μg·g^−1^), reflecting a resource trade-off between growth and defense metabolism. Genetic parameter analysis revealed that family-mean heritability (0.373–0.714) was higher than individual heritability and within-family heritability, with August identified as the optimal selection season. The maximum genetic gain across the three breeding strategies (individual selection, family selection, and combined selection) reached 7.86%, among which individual selection exhibited the smallest fluctuation in genetic gain. Finally, three superior families and 14 superior individuals were screened out. This study elucidates the seasonal genetic pattern of foliar CC in *P. taeda*, clarifies the optimal selection stage and efficient breeding strategies, and provides theoretical guidance and material support for the genetic improvement, germplasm innovation, and resource utilization of secondary metabolic traits in this ecologically and economically important tree species.

## 1. Introduction

Seasonal fluctuations in environmental factors frequently interfere with the growth and development of perennial trees throughout their life cycles [1]. To survive in such dynamically changing environments, trees have evolved intricate physiological and metabolic mechanisms to establish a dynamic physiological defense barrier [2,3]. As key “chemical weapons” enabling plants to adapt to and resist adverse conditions, secondary metabolites—including flavonoids such as catechin, proanthocyanidin, and quercetin—exhibit accumulation levels that are directly linked to plants’ chemical defense capacity [4,5,6,7]. However, constrained by internal resource availability and seasonal environmental variability, plants allocate fewer resources to defense when a greater proportion of resources is invested in growth during specific physiological periods [8]. This resource trade-off and precise allocation strategy between growth and defense drive pronounced seasonal dynamics and spatial heterogeneity in the synthesis and accumulation of metabolites [9]. Consequently, phenotypic evaluation at a single time point fails to accurately reflect the genetic potential of individual trees, which not only increases the difficulty of screening superior genetic resources in complex habitats but also constitutes a key bottleneck limiting tree genetic improvement [10,11].

It is well established that the physiological responses of forest trees to seasonal environmental changes are inherently governed by intrinsic gene regulation [12]. Heritability, a pivotal parameter in forest tree genetic improvement, serves as a core indicator for assessing the selection potential of target traits [13]. Previous studies have demonstrated that under fluctuating natural conditions, genotype–environment interaction (G × E) interactions elicit divergent genetic effects of the same genes across seasonal transitions [14,15,16]. Currently, however, assessments of the genetic potential of target traits in forest trees—such as secondary metabolite yield, stress resistance, and wood properties—remain confined to specific growth stages (e.g., seedling phase or growing season), with insufficient consideration of their genetic instability across seasons. This oversight has resulted in a notable neglect of the relationship between heritability and seasonal environmental variations [17,18]. Additionally, numerous studies have shown that fluctuations in water availability are often correlated with changes in flavonoid and phenolic acid content [19], with the composition of terpenoid volatiles in conifers exhibits distinct species specificity and is regulated by environmental factors [20,21]. Thus, exploring the variation patterns of genetic parameters and genetic effects of target tree traits across seasonal changes will facilitate the precise identification of superior forest germplasm, individual trees, and families with strong environmental adaptability, thereby further accelerating the pace of genetic improvement.

In recent years, the rapid development and widespread application of near-infrared spectroscopy (NIRS) technology have enabled large-scale, rapid, non-destructive, and high-throughput precise phenotyping of forest germplasm resources. This has laid a technical foundation for conducting dynamic trait monitoring and cross-seasonal, whole-life-cycle genetic evaluation of trees, effectively addressing the challenges of phenotypic evaluation posed by the spatiotemporal heterogeneity of metabolic traits [22,23]. For instance, Provaznik et al. [24] employed hyperspectral technology to perform needle phenotypic analysis across two seasons in a *Pinus sylvestris* clone seed orchard, verifying the operability and reliability of high-throughput phenotyping technology in the study of tree cross-seasonal traits. Furthermore, the best linear unbiased prediction (BLUP) model can effectively eliminate the random environmental interference, integrate individual phenotypic observations and family kinship information, accurately separate the environmental effects from individual phenotypic values, enable efficient estimation and precise dissection of the true genetic contribution (i.e., breeding value) of individual trees, and significantly improve the accuracy of genetic evaluation [25,26]. However, in breeding practice, individual selection strategies based on breeding values unilaterally pursues the maximization of short-term genetic gain for target traits, which is likely to result in the high concentration of selected superior individuals within a few core elite families, a sharp narrowing of the genetic base, an overly uniform population selection pattern, and reduced capacity to cope with climate change and biotic–abiotic stresses [27,28,29,30]. Therefore, how to steadily enhance the genetic gain of important genetic traits such as secondary metabolism, maintain the genetic diversity of the breeding population, and develop a balanced selection strategy that balances short-term benefits with long-term sustainability has become a core scientific problem urgently requiring resolution in the field of current precise tree breeding.

*Pinus taeda* L., an evergreen arbor of the genus *Pinus* in the family Pinaceae, is one of the most widely cultivated and highest-yielding industrial timber tree species worldwide, and also serves as a key pillar of wood production in forested areas of southern China [31,32]. Needles of *P. taeda* are rich in flavonoids such as catechin, which are natural bioactive raw materials with broad application potential in stress resistance, defense, and biomedicine [33,34]. However, existing cultivars exhibit low quality and limited quantities; moreover, traditional breeding has long focused on growth rate [35], wood quality [36], stem morphology [37], and disease resistance [38], leading to a paucity of research on secondary metabolic traits in needles that possess important physiological and economic value. Furthermore, the long-term lack of systematic analysis on the genetic rules governing catechin content (CC) across different seasons has hindered the conversion of needle resource advantages into breeding gains, thereby limiting the potential of *P. taeda* in comprehensive whole-plant improvement and production applications.

Thus, this study used 1697 individuals from 54 half-sib families of *P. taeda* as experimental materials. By integrating the NIRS model and BLUP model, we systematically analyzed the variation characteristics and genetic parameters of CC in *P. taeda* needles across different growing seasons, and evaluated the impact of different selection strategies on genetic gain. The main research objectives were as follows: (i) to analyze the dynamic variation characteristics of CC in *P. taeda* needles across different seasons; (ii) to estimate the genetic parameters of CC in different seasons and assess the impact of genotype × season (G × S) interactions on the accuracy of genetic evaluation; (iii) to compare the genetic gains of three breeding strategies—individual selection, family selection, and combined selection—and screen out elite materials with both high CC and genetic stability. The findings of this study will provide a scientific basis for the precise breeding of secondary metabolic traits in *P. taeda*, and also offer a reference for the multi-season genetic improvement of other perennial woody plants.

## 2. Results

### 2.1. Seasonal Dynamic Variation of Catechin Content

**Figure 1:**
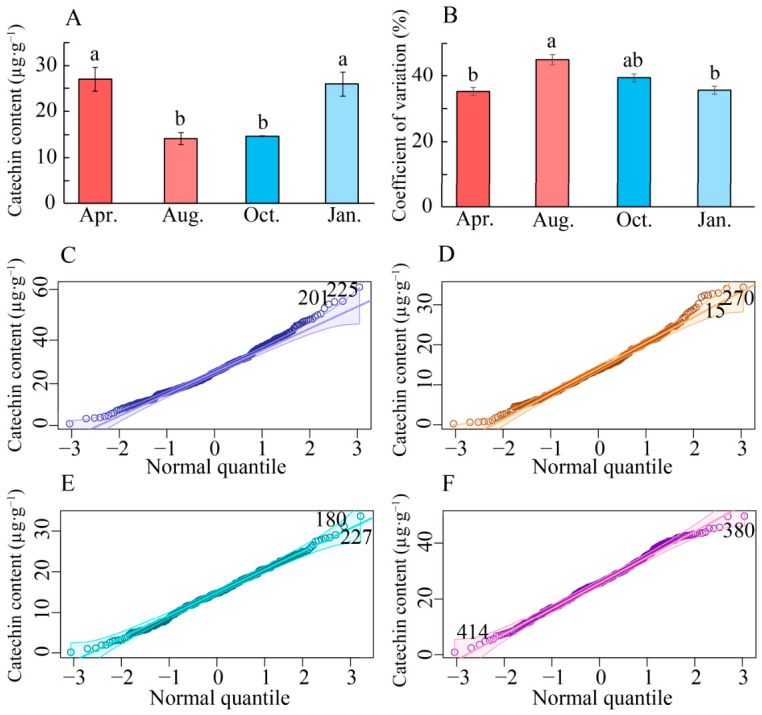
Seasonal dynamics of catechin content (CC) in needles of *P. taeda*. (**A**): CC (mean ± standard error) in each season. (**B**): Coefficient of variation in CC in each season. (**C**–**F**): Q–Q normal distribution plots of CC in each season. Different lowercase letters in the figure indicate significant differences in CC among seasons at the 0.05 level (*p* < 0.05).

Two-way analysis of variance (ANOVA) showed that both season and family exerted extremely significant main effects on needle catechin content (CC) in *P. taeda* needles (*p* < 0.001), with season being the dominant source of phenotypic variation in CC. The season × family interaction effect was not statistically significant (*p* = 0.101), indicating that the genetic expression of CC is stable across season and that the relative genetic ranking of families is not significantly affected by seasonal fluctuations (Table 1). Needle CC in *P. taeda* exhibited a bimodal accumulation pattern, with higher levels in spring and winter, and lower levels in summer and autumn. Specifically, CC in April (26.95 μg·g^−1^) and January (25.94 ± 0.45 μg·g^−1^) was extremely significantly higher than that in August (14.15 ± 0.39 μg·g^−1^) and October (14.65 ± 0.41 μg·g^−1^) (*p* < 0.001) (Figure 1A). Analysis of the coefficient of variation for CC across seasons showed that the inter-family variation was higher in August and October, suggesting greater selection potential during these periods (Figure 1B). In addition, the CC data in each season were normally distributed (Figure 1C–F), supporting the validity of subsequent genetic parameter analyses.

### 2.2. Family Genetic Variation and Seasonal Accumulation Characteristics of Catechin Content

**Figure 2:**
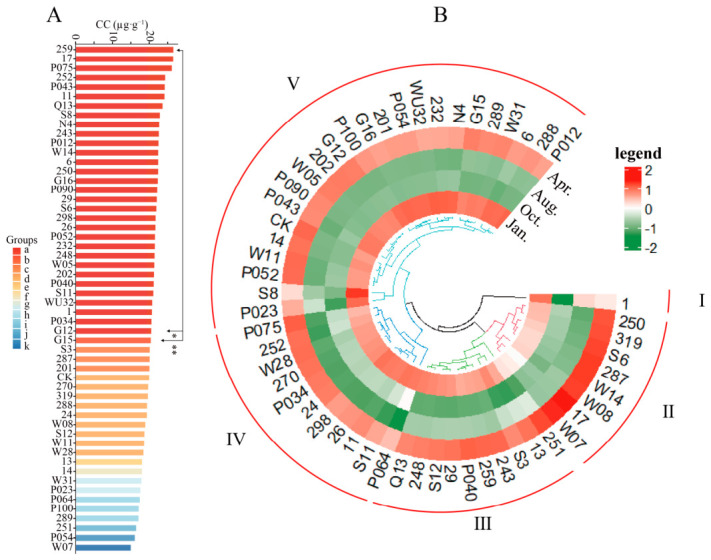
Family differences and cluster analysis of catechin content in *P. taeda*. (**A**): Duncan’s multiple comparisons of catechin among different families; differences between groups are labeled with lowercase letters a–k (*p* < 0.05), * indicates *p* < 0.05, and ** indicates *p* < 0.01. (**B**): Circular clustering heatmap of catechin content across four seasons.

There were significant genetic differences in needle CC among *P. taeda* families (*p* < 0.05) (Figure 2). The average CC of family 259 was significantly higher than that of family G15 (*p* < 0.05). Ten elite families were selected at a 20% selection intensity, with an average CC of 24.23 μg·g^−1^—17.41% higher than the population (20.64 μg·g^−1^). To further analyze differences in CC seasonal accumulation characteristics among families, hierarchical cluster analysis was performed on 54 families using data from all four seasons. The results showed that all families could be divided into five clusters with significant seasonal dynamic characteristics (Figure 2). The main characteristics and breeding application directions of each cluster are as follows: Cluster I (1 family): stable content type, with consistent CC throughout the year and the smallest seasonal fluctuation range; Cluster II (8 families): April-peak type, where CC reached the annual maximum in April (29.64 μg·g^−1^) and declined sharply in August and October, making it suitable for harvesting and utilization in April; Cluster III (10 families): August-dominant type, with significantly higher CC than other clusters in August and the annual minimum in October; Cluster IV (11 families): October-dominant type, with the highest CC among all clusters in October and the lowest in August; Cluster V (24 families): year-round elite type, where CC peaked in January (27.66 μg·g^−1^), had a seasonal coefficient of variation of only 26.1%, and exhibited both high CC and excellent seasonal stability.

### 2.3. Seasonal Dynamics of Heritability and Screening of Elite Families Across Seasons

Genetic parameter estimation revealed seasonal variations in the family-mean heritability, individual heritability, and within-family heritability of CC (Table 2). Among these, family-mean heritability was highest in summer and lowest in winter. Across the four seasons, family-mean heritability of CC was consistently higher than both individual heritability and within-family heritability. The BLUP model was used to estimate family effect values for each season, and the top 10 superior families based on effect values were screened for each season (Table 3). To obtain family materials with stable and high CC across seasons, a screening criterion was established: families must rank among the top 10 in family effect values for at least three seasons. Finally, three superior families with excellent overall performance were identified, namely P075, Q13, and 11.

### 2.4. Regulation of Family Background on Estimated Breeding Values

**Figure 3:**
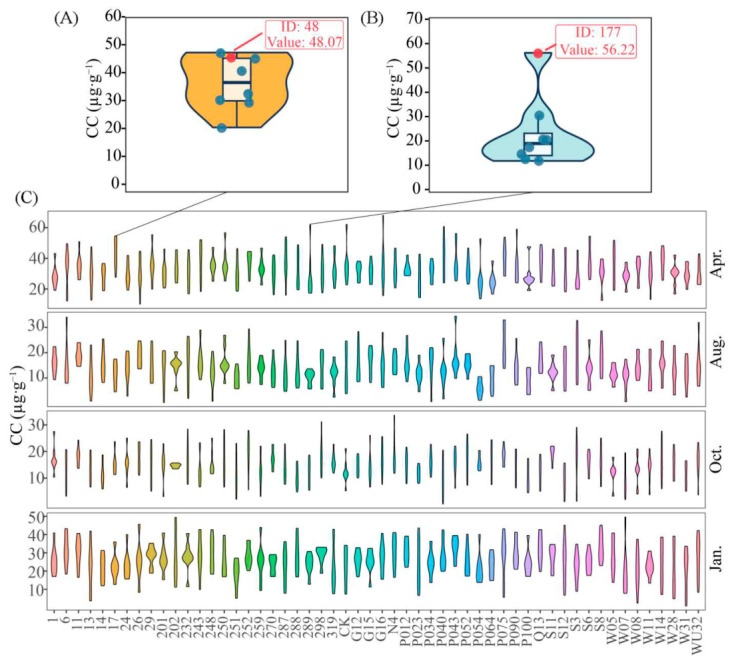
Seasonal distribution characteristics of CC across different family lineages. (**A**,**B**): Individual distribution characteristics of two superior families (Family 17 and Family 289) with prominent CC in *P. taeda* needles in spring, highlighting two individuals (ID: 48 and ID: 177) with outstanding measured CC values. (**C**): Intra-family distribution patterns of needle CC in *P. taeda* across different seasons, with each violin-box plot representing one family.

Figure 3 illustrates that the relationship between individual phenotypic values and predicted breeding values of needles CC in *P. taeda* is influenced by family background. For instance, individual 177 (from family 289) had the highest phenotypic value (56.22 μg·g^−1^), which was 1.17 times that of individual 48; however, its breeding value (2.71 μg·g^−1^) was substantially lower than that of individual 48 (breeding value 7.86 μg·g^−1^, family 17).

### 2.5. Screening Strategies for Optimizing Gain and Diversity

To achieve precise genetic improvement of CC in *P. taeda* and balance the maximization of genetic gain (GG) with the maintenance of family diversity, three elite tree selection strategies (SS) accounting for both genetic gain and family diversity were designed in this study, as follows: (1) Single-trait Breeding Value Selection (SE): Elite individuals were selected directly in descending order based solely on individual breeding values, without considering family number or selection proportion. (2) Family-Constrained Selection (FCS): Individual breeding value was still used as the selection criterion, but an upper limit of two selected individuals per family was imposed, to control the distribution of selected individuals and avoid excessive concentration of elite individuals in a few families. (3) Combined Selection Strategy (CSS): First, elite families were preliminarily selected, and 1–2 individuals with high breeding values were chosen within each selected elite family. Subsequently, 1 elite individual was supplementarily selected from each family with a family breeding value higher than the population average but not included in the elite families, ultimately forming the selected population.

**Figure 4:**
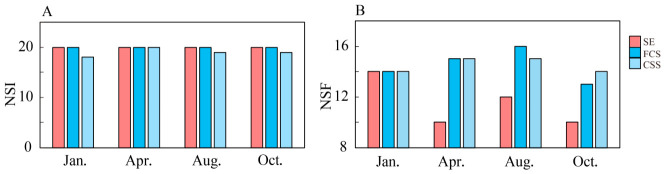
Selection performance of different screening strategies across different seasons. (**A**): Number of superior individuals and families selected by each strategy in different seasons; (**B**) Co-occurrence rate of selection results between each pair of the three screening strategies in different seasons. NSI: number of selected individuals; NSF: number of selected families; SE: Single Breeding Value Selection; FCS: Family-Constrained Selection; CSS: Composite Screening Strategy.

Table 4 shows the range of expected GG under different selection strategies. The maximum GG across the three strategies was 7.8557%, while the minimum values varied among strategies. Specifically, the GG range for SE was 1.9918–7.8557%, whereas the GG ranges for FCS and CSS were both 1.6013–7.8557%. The lower limit of GG for SE was slightly higher than those for FCS and CSS. In addition, the number of selected elite individuals remained stable at 18–20 plants across different sampling seasons (Figure 4A). Notably, the SE strategy resulted in the fewest family types among the selected elite individuals, with only 10 families contributing to elite selections in August and January. In contrast, the FCS and CSS strategies involved 13–16 families, with 15 families contributing to elite selections in August, representing a 50% increase compared with the SE strategy (Figure 4B).

### 2.6. Screening of Elite Individual Plants

Based on the results of seasonal trait dynamic monitoring and genetic parameter estimation, an elite individual screening scheme was established with summer as the core selection window. Our results showed that traits heritability in the summer was significantly higher than that in other seasons, confirming summer as the optimal selection period. For regulating family contribution, individuals selected via the balanced selection strategies (FCS and CSS) were used to control the family origin the distribution of the selected individuals. By comprehensively integrating seasonal dynamic characteristics, family seasonal response clustering, and multi-strategy comparison results, we constructed an elite individual screening process that incorporates these key elements. Ultimately, 14 elite individuals with the best and most stable performance were finally identified from 432 individuals (Table 5). Notably, all selected individuals passed the screening of three methods (SE, FCS and CSS), indicating that these 14 individuals possess advantages in both breeding value and family representativeness.

## 3. Discussion

To cope with seasonal environmental fluctuations, plants synthesize specialized secondary metabolites (such as phenols, terpenoids, and flavonoids) and modulate their metabolic abundance and profiles to strengthen defensive capacity [39,40]. Accumulation patterns of plant secondary metabolites are tightly coupled with ambient environmental stress conditions [41]. For instance, in winter and spring, coniferous trees often maintain high levels of phenolic compounds to resist stresses such as low temperature and strong ultraviolet radiation [42,43]. In contrast, the abundance of flavonoids (e.g., rutin and dihydroquercetin) decreases significantly during the vigorous growing seasons of summer and autumn [44]. Consistent with previous findings in *P. chinensis* [45], our results revealed pronounced catechin accumulation in *P. taeda* needles during winter (January) and spring (April). This seasonal pattern is likely attributed to the low temperatures prevailing in Guangdong during these periods, whereby plants accumulate abundant secondary metabolites to enhance antioxidant activity, photoprotection and cold tolerance [46]. During the vigorous growth stages of (August) and autumn (October), however, plants preferentially allocate limited photosynthates and carbon resources to primary metabolism processes, thereby suppressing catechin biosynthesis and reducing its accumulation [47,48]. Such seasonal rhythmicity of catechin accumulation, which is tightly synchronized with environmental stress intensity and plant growth cycles, provides robust evidence supporting the plant growth–defense trade-off theory [49].

Quantitative genetics theory indicates that specific environmental stressors (e.g., temperature [50], photoperiod [51], and precipitation [52]) serve as critical selective pressure, which amplifies the physiological differences in environmental responses among different genotypes and further increases the proportion of additive genetic variance ($\sigma_{A}^{2}$) in the total phenotypic variance [53]. In pine genetic assessments of growth and secondary metabolite biosynthesis, genetic parameters are strongly modulated by seasonal fluctuations in environmental factors [54]. For example, the total phenolic content of *P. elliottii* needles [55], the branch growth of *P. pseudostrobus* [56], the height growth of *P. sylvestris* [57], and the needle color [58] are all significantly regulated by seasonal environmental changes. In this study, the study detected distinct seasonal specificity in the genetic parameters of *P. taeda* needle CC, with both family-mean and individual heritability peaking in August. Combined with the highest coefficient of variation (*CV* = 45.09%) observed in August, these indicated that the high-temperature and strong-radiation conditions in August impose strong natural selection pressure on the experimental population. This may be attributed to August being the season of high temperatures and intense radiation in southern China, where significant environmental changes triggered differential expression of genes encoding pathways responding to abiotic stress signals and key enzymes involved in secondary metabolite synthesis in plants [59]. This pressure fully induced and amplified the physiological differences in catechin synthesis among different families, leading to the maximum release of additive genetic variance within the population.

While our study revealed pronounced seasonal variation in the genetic parameters of CC in *P. taeda*, genetic parameter estimation in forest trees inherently reflects the population genetic structure characterized under specific spatiotemporal conditions, with its accuracy inevitably constrained by multiple extrinsic and intrinsic factors. Key confounding variables include experimental population size [60], field experimental design [61], and individual developmental stage [62], all of which can bias the estimation of genetic variance components. Therefore, to overcome the limitations of single-time-point genetic evaluation, this study established a dynamic assessment framework based on continuous cross-seasonal monitoring. This approach enables comprehensive capture of genetically based variation in catechin accumulation in response to divergent seasonal environmental pressures, thereby providing more robust empirical evidence to support the early selection and targeted breeding of secondary metabolic traits in *P. taeda*.

As is well known, forest trees are inherently characterized by prolonged growth cycles, complex trait development, challenges in early-stage selection and high breeding investment costs [63,64,65,66]. Individual phenotypic performance represents the integrated outcome of intrinsic genetic potential and extrinsic microenvironmental variation [15], serving as the core evaluation index for forest tree genetic improvement. However, conventional phenotypic selection may misattribute transient environmental fluctuations or non-heritable heterosis to stable genetic effects, resulting in inaccurate breeding value estimation [67,68,69]. To precisely quantify the intrinsic genetic potential of individual trees, the BLUP model was employed in this study. By integrating pedigree and family background information, this approach corrects for stochastic environmental disturbances and eliminates estimation biases caused by phenotypic outliers [70,71,72]. The efficacy of this model has been verified in *P*. *tabuliformis* and other tree species, as it effectively balance selection intensity and prediction accuracy and enable screening and elimination of inferior individuals [73]. In the present study, comparative analysis of *P. taeda* families with divergent phenotypic distributions (exemplified by family 17 and family 289) revealed that extreme phenotypic values occurring in populations with low overall means and scattered distributions generally harbor lower genetic merit than average individuals from stable, high-performing populations. This discrepancy arises because the BLUP model identifies the extreme performance of family 289 as unreliable environmental noise, whereas the consistently superior phenotypic performance of family 17 is assigned higher genetic reliability.

Although the BLUP model enables high-precision breeding value estimation [29,74], selection based solely on phenotypic ranking carries a high risk of excessive concentration of selected individuals within a limited number of elite families [75]. Moreover, unconstrained intensive selection tends to cause a sharp reduction in effective population size by overrepresentation of a small set of superior families, thereby elevating inbreeding risk and weakening long-term genetic improvement potential of breeding populations [28,76,77]. In the present study, unconstrained single-strategy selection resulted in severe clustering of selected individuals within a few superior families; for instance, the superior family P075 alone accounted for nearly 30% of all selected candidates, consistent with previous observations in *P*. *koraiensis* [78] and *P*. *sylvestris* [79]. To mitigate this limitation, we implemented a balanced selection strategy by imposing a maximum family contribution. This approach expanded the number of retained families in the selected population to 13–16, while restricting the maximum contribution of any single family to 10% (a maximum of two selected individuals per family). This strategic transition gain-oriented selection to balance between genetic gain and genetic diversity is essential for maintaining the number of unrelated families and ensuring the long-term evolutionary potential of breeding populations [80]. Essentially, this strategy achieves environmental buffering by exploiting complementary seasonal responses response profiles across diverse families, offsetting productivity declines under suboptimal growing conditions at the expense of a minor reduction in short-term genetic gain [81,82]. For example, the high-yielding family P075 exhibited strong seasonal sensitivity, with substantial declines in catechin accumulation occurring in January. By limiting its selection proportion and incorporating families with stable cross-seasonal performance, balanced selection effectively mitigated overall phenotypic fluctuations in the population. This diversity-driven buffering mechanism has been verified in multi-environment stability analyses of conifers such as *P*. *pinaster* [83] and *P*. *sylvestris* [84]. Collectively, the three superior families (P075, Q13, and 11) and 14 elite individuals identified in this study integrate high catechin yield potential with strong environmental adaptability, providing valuable germplasm resources for future secondary metabolic traits and multi-generational crossbreeding programs in *P*. *taeda*.

Nevertheless, this study was performed within a single stand environment and exclusively focused on catechin as the target trait, which imposes certain limitations on the broader applicability of our conclusions. Future work will incorporate multi-site trials across distinct climatic zones and environmental conditions to validate and generalize the present findings. Furthermore, the rapid advancement of smart breeding and artificial intelligence (AI) technologies has substantially improved the precision and efficiency of plant genomic selection and phenotypic prediction [85]. Building on the BLUP-based genetic evaluation framework established in this study for *P. taeda*, further research will integrate genomic profiling and AI-assisted visual breeding tools to further enhance the predictive accuracy of key secondary metabolic traits. This integrated analytical pipeline will enable systematic dissection of G × E interaction effects and their underlying molecular regulatory mechanisms. Additionally, such advances will facilitate the optimization of multi-trait coordinated selection strategies, laying a robust scientific foundation for the comprehensive genetic improvement of *P. taeda* and other commercially and ecologically important forest tree species.

## 4. Materials and Methods

### 4.1. Test Site and Materials

**Figure 5:**
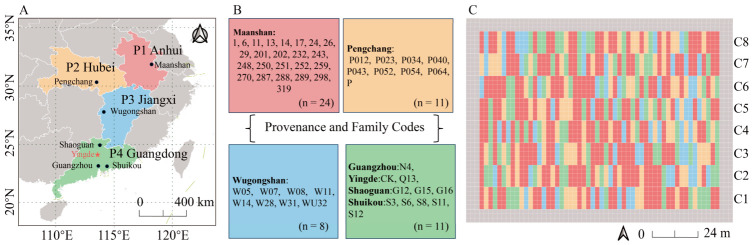
Provenance distribution and field planting layout of the experimental materials. (**A**): Geographical distribution of the seven provenances. (**B**): Abbreviation codes for the tested families derived from the seven provenances. (**C**): Block arrangement of the experimental materials in the field trial. The experimental materials consisted of families originating from four provincial provenances, including Anhui (P1, red, n = 24), Hubei (P2, yellow, n = 11), Jiangxi (P3, blue, n = 8), and Guangdong (P4, green, n = 11). The field trial was established with eight experimental blocks (C1–C8), with the longitudinal direction aligned to the north (N). The peripheral gray areas around indicate three protection rows.

The experimental site was located at the National Improved Seed Base of *P*. *taeda*, affiliated with the Yingde Forestry Research Institute in Guangdong Province, China (24°15′ N, 113°25′ E), with detailed information on the base provided in Table 6. Plant materials comprised 54 open-pollinated half-sib families derived from the 1.5-generation *P. taeda* seed orchard. The parental origins included superior provenances collected from Guangdong, Hubei, Jiangxi, and Anhui provinces in China (Figure 5A,B). Afforestation was carried out in spring 2015 using 1-year-old seedlings, following a randomized complete block design consisting of 8 blocks. Each block contained all 54 families, and each family was planted in a single-row plot of five individuals with a spacing of 3 m × 3 m. Three border rows were established around the experimental stand to eliminate potential edge effects on measurement accuracy (Figure 5C).

### 4.2. Sample Collection and Processing

Sampling was conducted in spring (April 2022), summer (August 2022), autumn (October 2022) of 2022 and winter (January 2023) of 2023, respectively. All sampling procedures were conducted within four fixed experimental blocks (C1, C3, C5 and C7). Each block contained 54 families, and the first and second individual trees of each family were selected for sampling. Needles from newly lignified twigs were collected from three distinct orientations per tree and pooled to form a single composite sample. All collected samples were preliminarily screened to exclude individuals with damaged foliage, pest and disease infection, or abnormal moisture content. After quality filtering, a total of 1697 valid samples were obtained, including 428 spring samples, 426 summer samples, 416 autumn samples and 427 winter samples.

Following collection, needle samples were immediately cleaned to remove surface impurities and rapidly rinsed three times with deionized water. Samples were subsequently oven-dried at 65 °C until reaching a constant weight. The dried samples were ground using a high-speed grinder (25,000 r/min) (FW-100, Hebi Metallurgical Machinery Equipment Co., Ltd., Hebi, China) and passed through a 30-mesh standard sieve (pore size approximately 0.595 mm) to obtain homogeneous powder. Powder samples were stored hermetically in silica gel desiccators. Prior to spectral collection, samples were equilibrated for 24 h in a constant-temperature and constant-humidity laboratory (25 ± 2 °C, relative humidity 50% ± 5%) to minimize the influence of environmental fluctuations on spectral measurements.

### 4.3. Near-Infrared Spectroscopy Acquisition and Preprocessing

Diffuse reflectance spectra of samples were collected using a DA7200 near-infrared spectrometer (Perten Instruments AB, Stockholm, Sweden) over a scanning range of 950–1650 nm with a resolution of 5 nm and a spot diameter of 3.5 cm. Each sample was repacked into three independent loading replicates, with the instrument automatically performing three rotational scans per loading, yielding a total of nine spectra per sample. The average spectrum calculated from the nine replicates was used for subsequent analysis. Spectral preprocessing was implemented in the Unscrambler X 10.4 software (CAMO Software AS, Oslo, Norway). A combined pretreatment strategy of standard normal variate (SNV) transformation and the Savitzky–Golay first derivative was applied to eliminate instrumental noise, baseline drift and light scattering interference derived from heterogeneous sample particle size.

### 4.4. Near-Infrared Prediction Model

The NIRS prediction model was developed according to the procedure described Lu et al. [86], following three core procedures: (1) Sample preparation and division: Needle samples collected from 102 *P*. *taeda* individuals were randomly selected for model individuals. After excluding 8 outlier samples, the remaining 82 samples were used to build the prediction model, with additional 12 samples reserved for external validation. (2) CC detection: *P*. *taeda* needle catechin content was analyzed using liquid chromatography–mass spectrometry (LC-MS; API 5000, Sciex, Framingham, MA, USA), with chromatographic separation performed on an ACQUITY UPLC BEH C18 column (2.1 mm × 100 mm, 1.7 μm, Waters, Milford, MA, USA). (3) Model construction and validation: A predictive model was established using partial least squares (PLS) regression combined with the optimal spectral preprocessing method (first derivative + standard normal variate transformation, FD + SNV), incorporating 14 principal components. The model yielded a calibration coefficient (Rc) of 0.9696 and a root mean square error of calibration (RMSEC) of 1.3084 μg·g^−1^. The cross-validation correlation coefficient (Rv) was 0.8171, accompanied by an RMSECV of 3.1052 μg·g^−1^. External validation confirmed a correlation coefficient (R) of 0.8807 between predicted and measured catechin contents across the 12 validation samples.

### 4.5. Data Statistics and Analysis

#### 4.5.1. Phenotypic Data Statistics

All data visualization and basic statistical analysis were performed using R software (v4.4.2). Phenotypic parameters of CC, including the mean value, standard deviation, and coefficient of variation (*CV*), were calculated. The formula for *CV* calculation is presented as follows:

$$CV=\frac{SD}{\bar{x}}\times 100\%$$

where *SD* is the standard deviation, and $\bar{x}$ is the mean value.

#### 4.5.2. Analysis of Variance and Variance Component Estimation

Genetic parameters were estimated independently across four seasons to avoid confounding effects between seasonal environmental variation and genetic effects. For single-season analyses, experimental blocks were treated as spatial replicates. Each family contained two individuals per block across four blocks, yielding a total of eight individuals per family per season (2 trees × 4 blocks). All genetic parameters, including heritability and breeding values, were estimated using ASReml–R V4 [87]. The BLUP model was employed to estimate family effect values and individual breeding values. Family-based and individual-based models were applied to calculate the individual narrow-sense heritability for each target trait, as described below:

Parent model:

$$\gamma_{ijk}=\mu +B_{i}+F_{j}+{BF}_{ij}+e_{ijk}$$

Single plant model:

$$\gamma_{ijk}=\mu +B_{i}+T_{ijk}+{BP}_{ik}+e_{ijk}$$

where *Y_ijk_* is the measured value of the trait for the *k*-th tree within the *j*-th family in the *i*-th block, *μ* is the overall mean, *B_i_* is the fixed effect of the *i*-th block, *F_j_* is the random effect of the *j*-th family, *BF_ij_* is the random interaction effect between the *i*-th block and the *j*-th family, assuming *BF_ij_*–*N*(0, *σ*^2^*BF*), *T_ijk_* is the random additive genetic effect of the *k*-th tree within the *j*-th family, *BP_ik_* is the random effect of the *k*-th plot within the *i*-th block, and *e_ijk_* is the random residual error, assuming *e*–*N*(0, *σ*^2^*_e_*).

#### 4.5.3. Heritability Estimation

In this study, all heritabilities refer to narrow-sense heritability (the ratio of additive genetic variance to phenotypic variance [73]), and the calculation formula is as follows:

Heritability of Single plant:

$$h_{S}^{2}=\frac{4\sigma_{F}^{2}}{\sigma_{F}^{2}+\sigma_{\mathrm{BF}}^{2}+\sigma_{E}^{2}}$$

Average heritability of family lines:

$$h_{F}^{2}=\frac{\sigma_{F}^{2}}{\sigma_{F}^{2}+\frac{\sigma_{BF}^{2}}{b}+\frac{\sigma_{E}^{2}}{nb}}$$

Heritability within families:

$$h_{w}^{2}=\frac{3\sigma_{F}^{2}}{\frac{\sigma_{BF}^{2}(b-1)}{b}+\frac{\sigma_{E}^{2}(bn-1)}{nb}}$$

where n represents the number of plants per plot, b represents the number of blocks, *σ*^2^*_F_* is the variance component of family effects, *σ*^2^*_BF_* is the variance component of block × family interaction effects, and *σ*^2^*_E_* is the variance component of error effects.

#### 4.5.4. Prediction of Genetic Effects and Estimation of Breeding Values

Genetic effects were estimated via the BLUP model, with the specific linear mixed model was constructed with reference to the method of Butler et al. [88]. In the model, trial site was specified as a fixed effect, whereas family, block, and family-by-block interaction were fitted as random effects. The family effect solutions derived from the model corresponded to the estimated breeding values (EBV) of each family.

#### 4.5.5. Estimation of Genetic Gain

The genetic gain was calculated according to the method of Falconer and Mackay [53], with the formula as follows:

$$∆G=h_{W}^{2}[\left(y_{ijk}-{\bar{y}}_{\cdot j\cdot }\right)+h_{F}^{2}({\bar{y}}_{\cdot j\cdot }-\hat{u})]$$

where *h*^2^*_W_* is the heritability within families, *h*^2^*_F_* is the family heritability, *y_ijk_* is the phenotypic value of the *k*-th individual plant in the *j*-th family of the *i*-th block selected, *ӯ_·j·_* is the mean value of the family where the *ijk*-th individual plant is located, and *û* is the estimated value of the test site mean.

## 5. Conclusions

Secondary metabolites are key modulators of plant environmental adaptability, growth and development, and evolutionary fitness. Here, this study integrated NIRS and BLUP modeling to systematically characterize the temporal dynamics and genetic characteristics of needle CC in *P. taeda*. Our results revealed a distinct seasonal rhythmicity in catechin accumulation, with significantly higher accumulation during winter and spring and diminished levels in summer and autumn. This seasonal variation aligns well with the canonical plant growth–defense resource trade-off hypothesis. Genetic parameter analysis showed that narrow-sense heritability of CC peaked in summer, indicating that enhanced seasonal environmental stress amplifies genotypic divergence in phenotypic expression. Consequently, summer constitutes the optimal seasonal window for the efficient genetic selection of catechin-associated traits in *P. taeda*. Furthermore, the constrained family contribution strategy based on BLUP evaluation expanded and increased the number of selected elite families by 30%—50% while maintaining robust genetic gain, effectively broadening the genetic diversity of the breeding population. Finally, three superior families (P075, Q13, and 11) and 14 elite individuals were screened based on catechin performance, providing elite germplasm for multi-generational crossbreeding and the development of high-value *P. taeda* germplasm resources. To further optimize the genetic evaluation system and advance precision breeding for superior *P. taeda* germplasm, future work will extend experimental coverage to multiple climatic regions and conduct long-term phenotypic monitoring. Combined with genomics and molecular biological techniques, subsequent studies will dissect the genetic regulatory characteristics and molecular mechanisms underlying key target traits, thereby providing a more robust theoretical foundation for the multi-generational genetic improvement of *P. taeda*.

## Figures and Tables

**Table 1:** Results of the variance analysis for family and seasonal effects. SV: source of variation; SS: sum of squares; df: degrees of freedom; MS: mean square; S × F: season × family interaction.

SV	SS	df	MS	F Value	*p* Value
Family	10,643	53	201	2.190	<0.001
Season	67,293	3	22,431	244.601	<0.001
S × F	16,843	159	106	1.155	0.101

**Table 2:** Estimation of genetic force across four seasons. FMH: Family-mean heritability; IH: Individual–tree heritability; WFH: Within–family heritability.

Month	FMH	IH	WFH
Apr.	0.576 ± 0.137	0.313 ± 0.155	0.272 ± 0.145
Aug.	0.714 ± 0.083	0.572 ± 0.189	0.537 ± 0.207
Oct.	0.514 ± 0.168	0.262 ± 0.159	0.225 ± 0.146
Jan.	0.373 ± 0.233	0.155 ± 0.145	0.130 ± 0.126

**Table 3:** Top 10 families ranked by the effect value of catechin content. The symbol ★ indicates that the family has ranked among the top ten in effect value for at least three seasons. CC: catechin content; FE: family effect.

Apr.			Aug.			Oct.			Jan.		
Family	CC (μg·g^−1^)	FE	Family	CC (μg·g^−1^)	FE	F	CC (μg·g^−1^)	F E	Family	CC (μg·g^−1^)	FE
17	38.15	9.0205	P075★	23.21	10.1596	S11	19.36	3.3334	S8	32.91	3.1427
P075★	34.64	5.7172	26	18.71	5.1701	252	19.00	3.0840	Q13★	32.47	2.9412
P040	33.89	5.5774	S3	18.59	5.0313	11★	18.11	2.4686	6	31.53	2.7877
243	32.75	4.6510	243	18.49	4.7438	26	17.92	2.3313	P043	32.83	2.7777
252	32.30	4.6246	11★	18.02	4.2207	P075★	18.25	2.0418	P012	30.97	2.2581
S6	33.13	4.5252	S8	17.71	4.0548	270	17.24	1.8674	N4	30.82	2.1918
W14	32.58	4.5137	1	16.13	3.4352	P052	17.24	1.8664	11★	30.30	1.8416
Q13★	32.11	4.1314	Q13★	16.95	3.2129	1	17.14	1.7940	P090	29.90	1.7731
287	31.94	3.8616	G15	16.78	3.0297	G16	16.98	1.6866	248	29.24	1.4740
P043	31.53	3.6640	250	16.42	2.6228	P064	16.53	1.3735	29	28.22	1.4477

**Table 4:** Selected results of superior individual plants under three screening strategies. ▲: SE; ●: FCS; ◆: CSS; F: family; ID: individual identity; CC: catechin content; GG: genetic gain; SS: selection strategy.

Apr.					Aug.					Oct.					Jan.				
ID	F	CC (μg·g^−1^)	GG (%)	SS	ID	F	CC (μg·g^−1^)	GG (%)	SS	ID	F	CC (μg·g^−1^)	GG (%)	SS	ID	F	CC (μg·g^−1^)	GG (%)	SS
48	17	48.07	7.8557	▲●◆	302	P075	32.95	8.815	▲●◆	137	252	27.87	3.0373	▲●◆	15	6	43.22	2.9691	▲●◆
47	17	46.57	7.5946	▲●◆	301	P075	32.33	8.5658	▲●◆	133	252	24.2	2.8559	▲●◆	10	6	41.93	2.7312	▲●◆
263	P040	55.23	7.5471	▲●◆	300	P075	30.26	7.8543	▲	136	252	24.48	2.7087	▲	363	S8	38.93	2.6183	▲●◆
104	243	45.02	6.2045	▲●◆	103	243	32.42	7.2618	▲●◆	329	S11	21.95	2.5769	▲●◆	365	S8	45.17	2.4435	▲●◆
301	P075	48.19	5.4041	▲●◆	299	P075	23.17	7.1757	▲	332	S11	21.97	2.4392	▲●◆	225	N4	40.65	2.3582	▲●◆
26	P040	42.77	5.3068	▲●◆	344	S3	32.74	7.097	▲●◆	6	1	27.47	2.4265	▲●◆	16	6	38.46	2.3499	▲
300	P075	39.56	5.0654	▲●◆	419	WU32	31.93	5.9793	▲●	20	11	24.22	2.3914	▲●◆	302	P090	41.32	2.2205	▲●◆
103	243	41.24	5.0642	▲◆	101	243	28.95	5.6566	▲●◆	130	252	21.16	2.3536	▲	167	288	43.45	2.2126	▲●◆
356	S6	48.86	5.0379	▲●◆	64	26	23.42	5.4653	▲●◆	401	W14	27.61	2.3406	▲●◆	326	Q13	35.74	2.1671	▲●◆
271	P043	50.64	4.8809	▲●◆	345	S3	27.65	5.4214	▲●◆	279	P052	26.51	2.3042	▲●◆	362	S8	31.62	2.1451	▲
159	287	43.85	4.8795	▲●◆	349	S3	22.11	5.4166	▲	60	26	23.15	2.2942	▲●◆	321	Q13	35.45	2.1416	▲●◆
120	250	51.13	4.7627	▲●◆	298	P075	17.87	5.2804	▲	327	S11	20.69	2.2486	▲	271	P043	35.62	2.1415	▲●◆
355	S6	47.81	4.6848	▲●	359	S8	28.59	5.1036	▲●◆	97	232	28.4	2.2286	▲●	322	Q13	42.76	2.1362	▲
371	W05	46.21	4.6781	▲●◆	133	252	29.39	5.0909	▲●◆	119	250	28.22	2.2159	▲●◆	319	Q13	42.46	2.1094	▲
160	287	48.41	4.6294	▲●	297	P075	15.73	4.8982	▲	328	S11	20.96	2.1456	▲	17	11	40.71	2.0773	▲●◆
325	Q13	39.12	4.6293	▲●◆	62	26	21.16	4.6592	▲●◆	343	S3	29.05	2.1402	▲●	103	243	42.76	2.0665	▲●◆
309	P090	53.45	4.5353	▲●◆	206	G12	28.35	4.4197	▲●	331	S11	20.62	2.094	▲	23	11	38.77	2.0597	▲●
136	252	38.39	4.5349	▲●◆	117	250	26.9	4.3647	▲●◆	64	26	23.6	2.0746	▲●◆	266	P043	33.14	2.0464	▲●◆
73	29	49.88	4.5057	▲●◆	60	26	24.65	4.3153	▲	228	N4	24.92	2.0689	▲●◆	269	P043	33.14	2.0457	▲
401	W14	37.38	4.4632	▲●◆	232	P012	21.74	4.2792	▲●◆	223	G16	25.54	2.0626	▲●◆	234	P012	35.15	1.9918	▲●◆
105	243	46.59	4.3502	●	221	G16	28.01	4.2252	●	289	P064	24.34	2.0385	●◆	227	N4	36.25	1.9713	●
					238	P012	26.81	4.2021	●	295	P075	23.67	2.021	●◆	300	P075	43.17	1.9152	●◆
					3	1	21.14	4.1759	●◆	151	270	22.61	1.9951	●◆	97	232	40.62	1.8702	●◆
					21	11	23.97	4.0096	●◆	291	P064	24.23	1.9767	●	373	W05	40.85	1.8557	◆
					216	G15	20.47	3.7943	●◆	351	S8	24.99	1.9375	●	426	WU32	42.22	1.6505	◆
					90	232	26.45	3.7885	●◆	193	319	22.66	1.7285	◆	233	P012	31.23	1.6359	●◆
					18	11	23.54	3.6149	◆	22	11	18.9	1.7231	◆	68	29	35.26	1.6013	●
					326	Q13	19.08	3.5567	◆	179	298	22.63	1.7205	◆					
					276	P052	19.57	3.1562	◆										
					396	W14	24.6	3.1552	◆										

**Table 5:** Superior individuals of *P. taeda* screened based on multi-season dynamic evaluation and integrated strategies.

ID	Family	ID	Family
301	P075	363	S8
302	P075	225	N4
325	Q13	48	17
321	Q13	263	P040
20	11	344	S3
17	11	137	252
271	P043	329	S11

**Table 6:** Basic overview of the experimental site. CT: Climate type; ET: Extreme temperature; MAP: Mean annual precipitation; ASH: annual mean sunshine hours; FFP: frost-free period; AT_10_: annual accumulated temperature above 10 °C; LT: Landform type; STY: Soil type; EL: Elevation; SG: Slope gradient; ST: Soil thickness.

Climate Characteristics		Soil Characteristics		Main Understory Vegetation
CT	Subtropical monsoon climate	LT	Low hilly landform	*Imperata cylindrica*, *Eriachne pallescens*, *Dicranopteris linearis*, *Blechnum orientale*, *Lophatherum gracile*, *Lygodium japonicum* et al.
ET	39 °C/1 °C	STY	Medium–clayey lateritic red soil	
MAP	1699.1 mm	pH	5.2–6.7	
ASH	1631.7 h	EL	50 ± 3 m	
FFP	320 d	SG	15 ± 5°	
AT_10_	7576 °C	ST	>1 m	

## Data Availability

The data presented in this study are available upon request from the corresponding author. The data are not publicly available due to ethical reasons.
